# ST-Segment Elevation Secondary to Spontaneous Pneumomediastinum in the Setting of COVID-19 Infection: A Case Report and Literature Review

**DOI:** 10.7759/cureus.25399

**Published:** 2022-05-27

**Authors:** Rafail Beshai, Peter Bulik, Hafeza Shaikh

**Affiliations:** 1 Internal Medicine, Jefferson Health - New Jersey, Stratford, USA; 2 Cardiology, Virtua Health, Camden, USA

**Keywords:** st elevations, spontaneous pneumomediastinum (spm), pneumomediastinum, covid-19, covid

## Abstract

A 45-year-old male presented with shortness of breath, cough,and chest discomfort. He reported positive test results for coronavirus disease 2019 (COVID-19) four days prior; this was confirmed by a second test administered at the hospital*.* Results of a chest CT, consistent with COVID-19 pneumonia, also revealed pneumomediastinum (PM). EKG showed ST elevations in the inferior leads with no reciprocal changes. Emergent cardiac catheterization showed that he had no stenosis in his major coronary arteries. His symptoms resolved after 25 days of hospitalization and the patient was ultimately discharged. This case highlights the importance of recognizing spontaneous PM as a complication of COVID-19 along with its uncommon presentation of ST elevation in order to prevent unnecessary invasive procedures.

## Introduction

Spontaneous pneumomediastinum (PM) is a rare complication seen in patients afflicted with a severe form of coronavirus disease 2019 (COVID-19). In rare cases, PM may mimic ST-elevation myocardial infarction (STEMI) on EKG. Clinicians should be aware of this unique concurrence to reach a proper diagnosis and provide appropriate management as well as prevent unnecessary invasive procedures.

## Case presentation

A 45-year-old male with a known past medical history of hypertension and a positive COVID-19 test four days prior presented to the emergency department with shortness of breath, cough, and chest discomfort. He reported that symptoms had started nine days prior and were getting progressively worse. He described his chest discomfort as chest heaviness without chest pain. All his symptoms were exacerbated by exertion. His pulse Ox O_2_ reading was 88% with a transient decline to 85% upon ambulating. He was started on 2 L of nasal oxygen via cannula. The physical exam showed crepitus upon palpation in his chest. Lung exams were significant for crackles at the bilateral bases. The heart exam showed no murmurs, rubs, or gallops. No friction rub, pulsus paradoxus, or jugular venous distention (JVD) was appreciated on the physical exam. The patient was not vaccinated against COVID-19.

**Figure 1:**
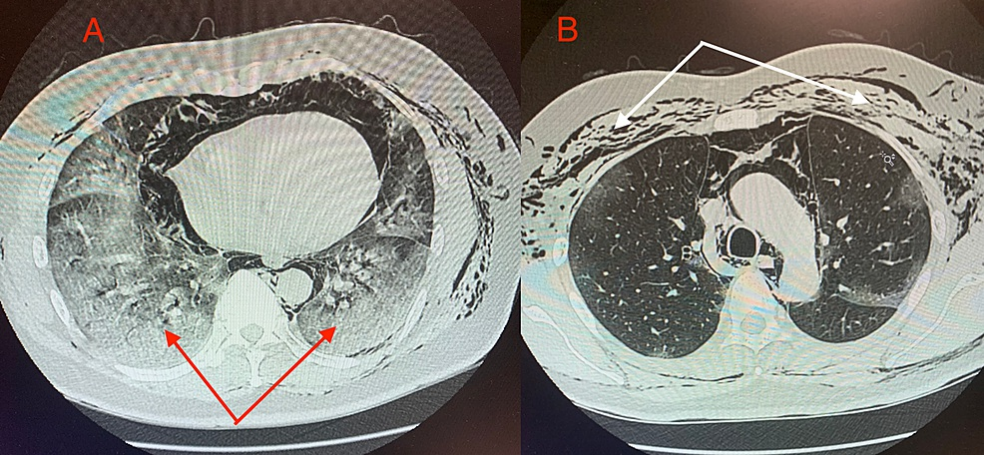
Coronal lung CT scan A) The image shows ground-glass air space opacities throughout bilateral lung bases consistent with COVID-19-positive pneumonia as shown by the red arrows. B) The image shows subcutaneous emphysema involving the right and left chest in a symmetric fashion as shown by the white arrows CT: computed tomography; COVID-19: coronavirus disease 2019

**Figure 2:**
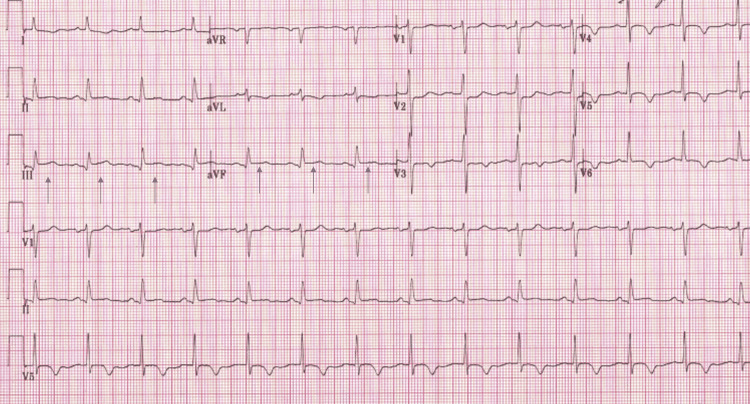
Twelve-lead EKG showing ST elevation in leads 3, and AVF The arrows show ST elevations EKG: electrocardiogram

**Figure 3:**
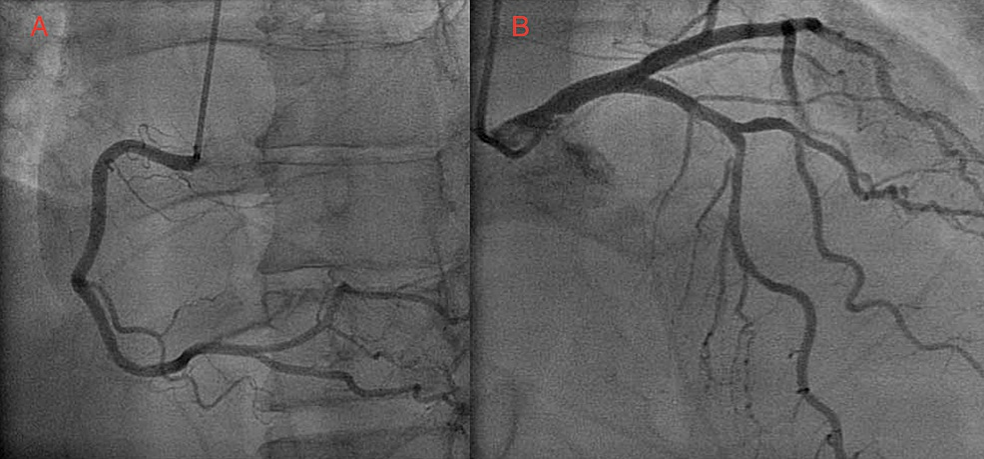
Left heart catheterization A) Left heart catheterization showing normal right coronary artery. B) Left heart catheterization showing normal left coronary artery

Another COVID-19 test administered at the hospital was also positive. A chest CT scan showed extensive ground-glass airspace opacities throughout both lungs, lower lobe-predominant, consistent with COVID-19-positive pneumonia (Figure 1A). In addition, it showed subcutaneous emphysema involving the right and left chest in a symmetric fashion due to PM (Figure 1B). CT angiogram was negative for pulmonary embolism. EKG performed in the emergency department showed ST elevation in the inferior leads with no reciprocal changes (Figure 2). Cardiac enzyme levels from laboratory tests performed showed slightly high sensitivity troponins at 36 ng/L and 45 ng/L (normal level: below 14 ng/L). Other labs were significant for erythrocyte sedimentation rate (ESR) of 20 mm/h (normal level: below 15 mm/h), and C-reactive protein (CRP) of 3.0 mg/dL (normal range: 0.8-1.0 mg/dL). A STAT transthoracic echocardiogram (TTE) showed normal wall motion with an ejection fraction of 60%. Even though the probability of acute coronary syndrome was low, cardiology decided to have the patient undergo emergent cardiac catheterization to rule out myocardial infarction as a reason for his ST elevations. It showed that he had no stenosis in his major coronary arteries (Figure 3).

The patient was given remdesivir 100 mg IV for five days for his COVID-19 infection. He also received azathioprine 50 mg twice daily due to interstitial changes noted at lung bases and dexamethasone 10 mg twice daily due to pulmonary involvement. In addition, he was encouraged to use incentive spirometry. After 25 days of close follow-up in the hospital, the patient’s symptoms including shortness of breath, chest discomfort, and cough finally improved, and a CT scan showed resolution of his PM. His ST elevations on EKG were found to have disappeared.

## Discussion

Acute myocardial infarction is the most clinically significant cause of ST-segment elevation; however, the differential diagnosis for ST elevation includes, but is not limited to, PM, pericarditis, and pulmonary embolism. Acute myocardial infarction was ruled out by cardiac catheterization that did not show any stenosis and TTE that did not show wall motion abnormalities. Even though the ESR and CRP levels were mildly elevated in our patient, the suspicion for pericarditis causing this ST elevation was very low given the lack of pericardial effusion on echocardiogram and, usually in pericarditis, there is diffuse ST elevation not limited to inferior leads like in our patient. Additionally, the patient's history was negative for any pleuritic chest pain and the physical exam did not show any signs of friction rub, pulsus paradoxus, or JVD. No pulmonary embolism was appreciated on the CT angiogram, making this diagnosis an unlikely one.

PM, also known as mediastinal emphysema, is a rare condition described as the abnormal presence of air or any other gas in the mediastinum. PM is classified into two categories; one, with an instigating event, is referred to as secondary PM, and the other occurs when free air is discovered in the mediastinal cavity without a clear etiology, and it is referred to as spontaneous PM [1]. Pulmonary barotrauma during invasive mechanical ventilation, endoscopic procedures, central line placements, and blunt face trauma are the main risk factors for secondary PM [2]. Tobacco use, chronic obstructive pulmonary disease, and asthma are the main risk factors for idiopathic spontaneous PM. However, spontaneous PM can also occur as a rare complication of infectious pneumonia, most commonly after staphylococcal, tuberculosis, or fungal pneumonia; however, it is relatively rare after viral pneumonia [2].

Recent publications have suggested an association between COVID-19 and PM. The association has been linked to the barotrauma from mechanical ventilation in these patients; however, there have also been increasing reports of spontaneous PM in the absence of mechanical ventilation [2-8]. The exact pathophysiology mechanisms of spontaneous PM in COVID-19 patients still remain unclear. However, the hypothesis is that there are some structural alterations in the lung parenchyma from COVID-19. These include surface protein disruption, such as downregulation of surfactant, loss of extracellular matrix and basement membrane, damaged type 2 pneumocytes, and hypercoagulability [9]. A recent multicenter retrospective case-control study showed that PM as a complication of COVID-19 was associated with a worse prognosis in terms of mortality and length of hospitalization [10].

To our knowledge, only a few case reports are available on PubMed and Google Scholar describing ST elevations secondary to PM (Table 1). We used the terms ‘pneumomediastinum’, ‘air block syndrome’, and ‘mediastinal emphysema’ with ‘ST elevations’ for our search. We believe that this is the first case to describe ST-segment elevation secondary to spontaneous PM in the setting of COVID-19 infection.

**Table 1:** Previous case reports describing ST elevations secondary to pneumomediastinum EKG: electrocardiogram

Author	Age in years/gender	Type of pneumomediastinum	Cause of pneumomediastinum	EKG findings	Cath lab findings
Macrae et al. [11]	26/M	Non-spontaneous	Intranasal insufflation of cocaine	ST-segment elevation in an inferolateral distribution	Did not go to the cath lab
Frenkel et al. [12]	56/M	Non-spontaneous (secondary)	Secondary to facial trauma and tracheostomy	ST-segment elevations in an inferior-lateral distribution	Balloon angioplasty was done but his ST elevation and chest discomfort persisted afterward
Sin et al. [13]	22/M	Non-spontaneous (secondary)	Barotrauma from mechanical ventilation	ST-segment elevations in an inferior-lateral distribution	Did not go to the cath lab
Lolay et al. [14]	46/M	Spontaneous (primary)	Started while helping his friend move his furniture	ST-segment elevation in the anterolateral leads with reciprocal changes in the inferior leads	Coronary angiography demonstrated no evidence of myocardial injury
Brearley et al. [15]	46/M	Non-spontaneous (secondary)	Barotrauma from mechanical ventilation	ST-segment elevation in the inferior-lateral leads	Did not go to the cath lab
Shukla et al. [16]	50/M	Non-spontaneous (secondary)	Right brachiocephalic vein thrombectomy	ST-segment elevation in the anterior-lateral leads	Normal coronary arteries

This review highlights the importance of considering the diagnosis of PM in patients with ST elevations. In our review, the age at the occurrence of the condition ranged from 22 to 56 years, with a 100% male preponderance. There were significant EKG findings in all the cases and 83% of all the cases had an EKG showing ST elevations in the inferior-lateral leads. Despite the EKG changes, coronary angiography demonstrated no evidence of myocardial injury in patients who went to the cath lab. Our review demonstrated different causes for PM leading to ST elevations. However, our report is the first to make the connection between PM and ST elevations secondary to COVID-19.

EKGs lack the necessary sensitivity and specificity to function as a sole diagnostic test for ischemia. Consequently, it must be interpreted based on the clinical context [17]. The exact etiology of how PM may lead to ST elevation is not clear and not well understood. It has been hypothesized that the ST elevations in PM are secondary to cardiac rotation or displacement, right ventricular enlargement, and the insulation of the heart by mediastinal air [14].

## Conclusions

This case report along with the literature review highlights the importance of identifying other etiologies of ST-segment elevation. It serves as a reminder of the importance of clinical correlation combined with both imaging and laboratory evaluation in patients with PM who present with ST-segment elevation in order to prevent unnecessary invasive procedures.
